# Antibiotic Resistance Profiling and Phylogenicity of Uropathogenic Bacteria Isolated from Patients with Urinary Tract Infections

**DOI:** 10.3390/antibiotics12101508

**Published:** 2023-10-03

**Authors:** Muhammad Ajmal Khan, Atta Ur Rahman, Bakhtawar Khan, Samiah Hamad Al-Mijalli, Amal S. Alswat, Aftab Amin, Refaat A. Eid, Mohamed Samir A. Zaki, Sadia Butt, Jamshaid Ahmad, Eman Fayad, Amin Ullah

**Affiliations:** 1 Centre for Biotechnology and Microbiology, University of Peshawar, Peshawar 25000, Khyber Pakhtunkhwa, Pakistan; ajmal.mb137@gmail.com (M.A.K.); jamshaidbiotech@yahoo.com (J.A.); 2 Leprosy Laboratory, Department of Parasite Biology, Oswaldo Cruz Institute, Oswaldo Cruz Foundation, Rio de Janeiro 21040-360, Brazil; atta25394@gmail.com; 3 Institute of Brain Disorders, Department of Physiology, Dalian Medical University, Dalian 116044, China; 4 Department of Biology, College of Sciences, Princess Nourah Bint Abdulrahman University, P.O. Box 84428, Riyadh 11671, Saudi Arabia; shalmejale@pnu.edu.sa; 5 Department of Biotechnology, College of Sciences, Taif University, P.O. Box 11099, Taif 21944, Saudi Arabia; a.alswat@tu.edu.sa (A.S.A.); e.esmail@tu.edu.sa (E.F.); 6 Center for Cancer Research, and State Key Lab of Molecular Neuroscience, Division of Life Science, Hong Kong University of Science and Technology, Hong Kong, China; aftabamin@ust.hk; 7 Department of Pathology, College of Medicine, King Khalid University, P.O. Box 62529, Abha 12573, Saudi Arabia; refaat_eid@yahoo.com; 8 Anatomy Department, College of Medicine, King Khalid University, P.O. Box 62529, Abha 61413, Saudi Arabia; mszaki@kku.edu.sa; 9 Department of Microbiology, Shaheed Benazir Bhutto Women University Peshawar, Peshawar 25000, Khyber Pakhtunkhwa, Pakistan; sadiabutt@sbbwu.edu.pk; 10 Department of Health & Biological Sciences, Abasyn University Peshawar, Peshawar 25000, Khyber Pakhtunkhwa, Pakistan

**Keywords:** urinary tract infections, uropathogens, phylogenetic tree, antibiotics, resistance

## Abstract

Urinary tract infections (UTIs) are healthcare problems that commonly involve bacterial and, in some rare instances, fungal or viral infections. The irrational prescription and use of antibiotics in UTI treatment have led to an increase in antibiotic resistance. Urine samples (145) were collected from male and female patients from Lower Dir, Khyber Pakhtunkhwa (KP), Pakistan. Biochemical analyses were carried out to identify uropathogens. Molecular analysis for the identification of 16S ribosomal RNA in samples was performed via Sanger sequencing. Evolutionary linkage was determined using Molecular Evolutionary Genetics Analysis-7 (MEGA-7). The study observed significant growth in 52% of the samples (83/145). Gram-negative bacteria were identified in 85.5% of samples, while Gram-positive bacteria were reported in 14.5%. The UTI prevalence was 67.5% in females and 32.5% in males. The most prevalent uropathogenic bacteria were *Klebsiella pneumoniae* (39.7%, 33/83), followed by *Escherichia coli* (27.7%, 23/83), *Pseudomonas aeruginosa* (10.8%, 9/83), *Staphylococcus aureus* (9.6%, 8/83), *Proteus mirabilis* (7.2%, 6/83) and *Staphylococcus saprophyticus* (4.8%, 4/83). Phylogenetic analysis was performed using the neighbor-joining method, further confirming the relation of the isolates in our study with previously reported uropathogenic isolates. Antibiotic susceptibility tests identified *K. pneumonia* as being sensitive to imipenem (100%) and fosfomycin (78.7%) and resistant to cefuroxime (100%) and ciprofloxacin (94%). Similarly, *E. coli* showed high susceptibility to imipenem (100%), fosfomycin (78.2%) and nitrofurantoin (78.2%), and resistance to ciprofloxacin (100%) and cefuroxime (100%). Imipenem was identified as the most effective antibiotic, while cefuroxime and ciprofloxacin were the least. The phylogenetic tree analysis indicated that *K. pneumoniae*, *E. coli*, *P. aeruginosa*, *S. aureus* and *P. mirabilis* clustered with each other and the reference sequences, indicating high similarity (based on 16S rRNA sequencing). It can be concluded that genetically varied uropathogenic organisms are commonly present within the KP population. Our findings demonstrate the need to optimize antibiotic use in treating UTIs and the prevention of antibiotic resistance in the KP population.

## 1. Introduction

Urinary tract infections (UTIs) are a common occurrence, primarily involving bacteria. However, in rare cases, fungal and viral infections have also been reported [1]. Bacteriuria, the presence of bacteria in the urine, can be classified as symptomatic (accompanied by UTIs) or asymptomatic [2]. UTIs, when left untreated, lead to systemic complications. Complications in kidney function owing to UTIs are associated with high morbidity and mortality [3,4]. In recent years, there has been an increase in the number of UTI cases [5]. UTIs are now the second most common type of infection in the body (urology care). People of all ages are affected by UTIs. However, women aged 16 to 35 are primarily affected [6]. Each year, 10% of women are diagnosed with UTIs (UTIs), while 60% will be infected at least once in their lifetime [6]. Anatomical differences have been attributed to the high incidence of UTIs in women compared to men [7]. Bacteriuria has often been linked to sexual activity. However, UTIs are not considered to be sexually transmitted diseases [8,9]. Moreover, obesity, diabetes and family history are key UTI determinants [10]. Pregnant women, in particular, are classified as a high-risk group [11]. Biochemical and physiological changes during pregnancy lead to an increase in urine amino acid and glucose concentrations, which promote bacterial growth [12]. Furthermore, childbirth also leaves mothers more susceptible to infections [13,14].

Upper urinary tract infections are referred to as kidney infections, while lower urinary tract infections are classified as bladder infections [15]. Both forms of infection have similar signs and symptoms in adults, such as frequent urination, an urge to urinate (despite having an empty bladder) or pain during urination [16]. Kidney infections are often associated with flank pain and high fever [17]. In severe cases, blood is also passed in the urine. In young patients, nonspecific symptoms may be present, including fever, while infants may develop poor eating habits, irregular sleeping habits and jaundice [18]. UTIs in the elderly are the most difficult to diagnose. Incontinence and the degradation of cognitive abilities become prominent.

Treatment involves the prescription of antibiotics. Antibiotic courses are mainly dependent on the medical history of the patient. Patients with a history of recurrent UTI infections are typically prescribed short antibiotic courses, which effectively treat UTIs and prevent antibiotic abuse [19]. Other patients are typically prescribed long antibiotic courses [19]. Cases involving antibiotic resistance are on the rise [20] and have been attributed to inappropriate antibiotic prescription and use. In such cases, stronger alternate antibiotics have to be used. However, this raises the possibility of developing antibiotic resistance against non-conventional agents. Healthcare professionals should responsibly prescribe specific antibiotics and inform patients of the risks of not completing prescribed treatment regimens. Nitrofurantoin and methenamine are examples of commonly prescribed antibiotics. The susceptibility patterns and genetic diversity of uropathogens in local populations must be monitored. In Pakistan, the acquired resistance of the community to UTI pathogens has not been thoroughly demonstrated. The cases of UTI and antibiotic resistance are spreading at an alarming rate in Pakistan, leading to increased morbidity and mortality.

The phylogenetic relationships among distinct bacteria can be investigated across various genomic regions using molecular and genetic research techniques. Due to high conservation and ease at which amplification can be carried out, distinct rRNA genes such as 16S rRNA are frequently utilized in comparative research to explore the phylogenetic diversity of various bacterial species [21,22]. The main benefit of this molecular method is that it is rapid, can be performed within hours, and the small differences amongst species can be determined. The 16S rRNA gene sequence comparison has become the genetic technique of choice for identifying differences and similarities between species. New species of bacteria can be identified, and the genetic relationship between isolates can also be determined, allowing closely related organisms to be classified [23]. The rapid and accurate identification of bacterial species is further made possible by developments in computer-aided techniques for biological data analysis [24,25]. In the case of uropathogens, the 16S rRNA method for bacterial identification has been employed in many studies [26,27,28,29]. Therefore, our study evaluated uropathogenic antibiotic susceptibility patterns and genetic diversity to facilitate the effective treatment and management of UTIs in patients from Lower Dir, Khyber Pakhtunkhwa, Pakistan.

## 2. Materials and Methods

### 2.1. Ethical Review Statement

The institutional bioethical committee (IBC) of Hazara University Mansehra, Khyber Pakhtunkhwa (KP), Pakistan, evaluated this research and found that it follows the university’s ethical principles and policies.

### 2.2. Specimen Collection and Study Duration

Experiments (1 November 2018–30 May 2019) were conducted in the Microbiology Section, Dr. Fazal Rahim Clinical Laboratory, Lower Dir, Pakistan. Urine specimens were collected from 145 patients within the community. These patients were suspected of having bacteriuria. During their routine checkup, patients were advised by their physicians to undergo tests. Midstream urine specimens (MSU, 20–30 mL) were collected in a sterile, wide-mouth, leak-proof urine container and labeled for microbiological examination.

### 2.3. Inclusion and Exclusion Criteria

Samples were obtained from people of all ages, genders and ethnic groups in this study. Positive cases were identified through diagnostic procedures: (1) Physical examination of urine samples, which involved the swirling or inversion of vials to examine the presence of turbidity or cloudiness. As an indicator, turbidity is associated with the presence of bacteria, proteins, crystals, leucocytes, urates (acids), phosphates and carbonates (alkalines), as well as the precipitation of these substances. (2) Real-time PCR facilities at the Dr. Fazal Rahim Clinical Laboratory were used. Negative cases were identified similarly and excluded (physical examinations that showed a lack of turbidity. Female patients were instructed to wash their urethral area with fresh water and dry themselves before MSU collection, while male patients were instructed to wash their hands thoroughly before MSU collection.

### 2.4. Specimen Processing

Specimens were mixed by inversion (2–3 times), streaked onto appropriately labeled MacConkey agar (Oxoid; exclusive for Gram-negative) and phenylethyl alcohol agar (PEA, exclusive for Gram-positive) media plates, and incubated at 37 °C in incubators with 5% CO_2_ for 24 h [30,31]. The plates were then examined macroscopically and microscopically for bacterial growth. Plates that had negative growth were re-incubated for another 24 h. The number of bacteria grown on the medium was reported as colony-forming unit per milliliter (CFU/mL). The serial dilution (10^5^) was used to calculate the CFU/mL with the following formula:

CFU/mL = (no. of colonies × dilution factor)/volume of culture plate in mL

The general standard criteria for UTI is a colony count of ≥104 cfu/mL as per the World Health Organization (WHO), considered a significant bacteriuria [32].

### 2.5. Identification of Isolates

Various biochemical tests were applied to differentiate Gram-negative bacteria from Gram-positive bacteria. Gram staining, catalase, coagulase, oxidase and SIM (Sulfur Indole Motility) testing were performed to differentiate diverse bacterial isolates [33]. The bacteria isolates were subcultured for antibiotic susceptibility testing.

### 2.6. Molecular Identification of Isolates

Pure bacterial cells (2 × 10^9^ CFU/mL) grown in nutrient agar medium were harvested and sent to the Beijing Genomics Institute Mainland China (BGI) (https://en.genomics.cn/ (accessed on 15 December 2022)) for 16S rRNA sequencing. The primers used for the amplification of the gene via polymerase chain reaction (PCR) were Forward 27F—5′-AGAGTTTGATCMTGGCTCAG-3′ and Reverse 1492R– 5′-CGGTTACCTTGTTACGACTT-3′ [27,34]. The PCR reaction mixture (25 µL) contained the following: 12.5 µL of PromTaq^®^Green Master Mix Promega, 1.25 µL of 27F primer, 1.25 µL of 1492R primer, 5 µL nuclease-free water and 5 µL template DNA. The PCR profile was as follows: initial denaturation at 94 °C for 5 min, denaturation at 94 °C for 60 s in 35 cycles, 45 s at 53 °C annealing temperature for 35 cycles and elongation step at 68 °C for 2 min in 35 cycles reaction. The final extension for the reaction was at 72 °C for 10 min and held at 4 °C. The PCR product was run on 1% agarose gel and TAE as a buffer and visualized with ethidium bromide staining. The PCR product’s expected size with primer is ±1465 bp. The automated DNA sequencer ABI 3730XL was used to sequence the PCR amplicon by the BGI.

The chromatograms obtained from BGI were analyzed using Chromas software 2.6.6 (http://technelysium.com.au/wp/chromas/ (accessed on 28 December 2022)) to trim the low-quality reads for further refinement. Nucleotide sequences were compared for similarity to 16S reference sequences using the Basic Local Alignment Search Tool (BLAST) algorithm, a National Center for Biotechnology Information (NCBI) database. The sequences for each bacterial isolate were submitted to NCBI GenBank for accession number.

### 2.7. Phylogenetic Analysis of Isolates

Phylogenetic network analysis of the bacterial isolates was performed using Molecular Evolutionary Genetics Analysis software 7 (MEGA version 7: http://www.megasoftware.net (accessed on 10 January 2023)). Sequence alignment was performed using a reference sequence, and neighbor-joining algorithms were used for phylogenetic tree construction using the default setting with 1000 bootstrap values [35,36,37,38].

### 2.8. Antibiotic Susceptibility Testing

Experiments were conducted according to the Clinical and Laboratory Standards Institute (CLSI) guidelines, using the Kirby–Bauer disk diffusion method to determine uropathogenic susceptibility [39,40]. Bacteria grown on Muller–Hinton Agar were exposed to different antibiotics using the disc diffusion (Kirby Bauer’s) technique. Cephradine (30 µg), imipenem (10 µg), meropenem (10 µg), gentamicin (10 µg), ciprofloxacin (5 µg), nitrofurantoin (300 µg), amikacin (30 µg), lincomycin (15 µg), cefotaxime (30 µg), fosfomycin (200 µg), clindamycin (2 µg), penicillin (10 µg), cefixime (10 µg) and cefuroxime (30 µg) disks were used in this regard.

### 2.9. Kirby–Bauer Disk Diffusion Method

The Kirby–Bauer disk diffusion method was used with slight modifications. This is a frequently used method in which an inoculum of the test bacterium (having 0.5 McFarland turbidity; 1.5 × 10^8^ CFU/mL) was streaked onto plates containing Muller–Hinton Agar (MHA) medium. MHA has many advantages. It supports the growth of all types of bacteria as it is a non-selective and non-differential medium. Loose agar facilitates efficient antibiotic diffusion. Starch helps absorb toxins released by bacteria, which may affect the inhibitory potential of antibiotics [41]. After the bacteria had grown, antibiotic discs were placed onto plates using sterile forceps to examine the respective susceptibilities of each bacterial species. Antibiotic discs were purchased from Bioanalyse, Ankara, Turkey. Bacteria were exposed to antibiotics for 24 h at 37 °C, and the zone of inhibition (ZOI) was measured in millimeters according to CLSI-M100 (2022) rules and regulations [39].

### 2.10. Statistical Analysis

Data were statistically analyzed using the chi-squared test using SPSS software (22.0 version) for comparisons. *p*-values greater than 0.05 were considered statistically significant.

## 3. Results

### 3.1. UTI Prevalence, concerning Gender and Age

**Figure 1:**
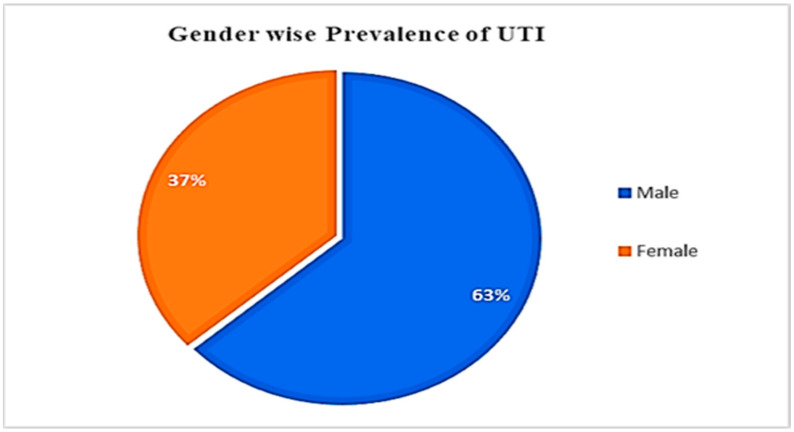
Distribution of UTIs in patients based on gender from the current study.

Specimens were collected from 145 patients with urinary tract infection (UTI) (Figure 1 and Supplementary Table S1). Most patients were of the ages 16–25 (20%) or 26–35 (18.62%). Elderly patients (ages 66 to 75 years; 7.59%) had the lowest UTI incidence amongst the study cohort. The correlation between the UTI patient gender and age was statistically insignificant (*p* = 0.114) (Table 1).

### 3.2. Morphological and Biochemical Identification of Uropathogens

After confirming the presence of uropathogens in patient samples, morphological and biochemical investigations were carried out to determine the presence of bacteria listed in Table 2. Colony characteristics were observed for various bacterial species: *K. pneumoniae* formed yellow to whitish colonies; *E. coli* formed yellow or opaque colonies; *P. aeruginosa* formed yellow, green, or blue colonies; *S. aureus* formed pale yellow colonies; *P. mirabilis* formed pale or colorless colonies; while *S. saprophyticus* formed cream to white colored colonies. Furthermore, biochemical assessments were also performed, involving Gram staining, catalase, coagulase, oxidase and Sulfur Indole Motility (SIM) tests. Bacteria were identified using Bergeys’ Manual of Systematic Bacteriology (Table 2).

### 3.3. Molecular Identification and Phylogenetic Network Analysis of Uropathogens

**Figure 2:**
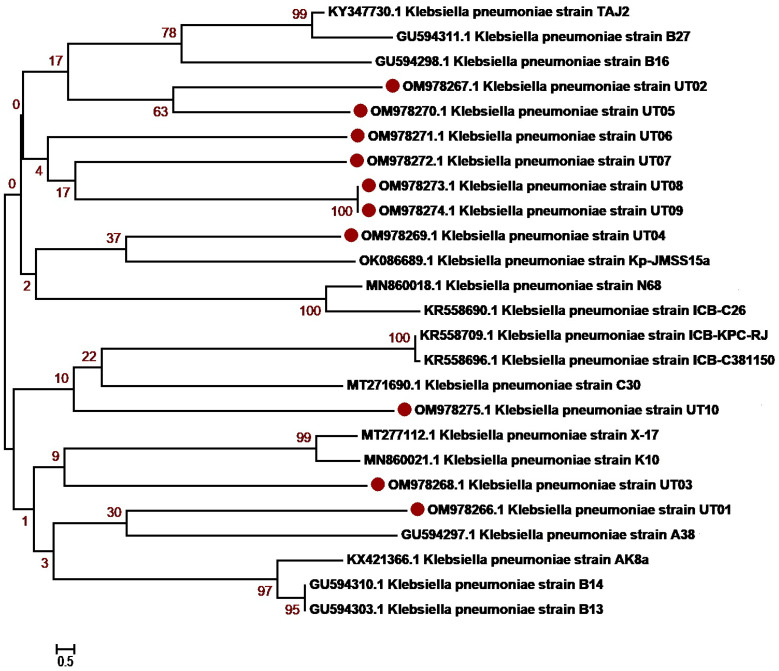
***Klebsiella pneumoniae* genetic diversity based on 16 S rRNA gene sequences with reference sequences.** The analysis includes 25 nucleotide sequences from GenBank, with the accession numbers shown in parentheses. The study sequences were identified with maroon dots in the figure. The percentage of replicate trees where the related taxa clustered together in the bootstrap test (1000 repetitions). MEGA7 software was used for sequence alignment and evolutionary analyses.

**Figure 3:**
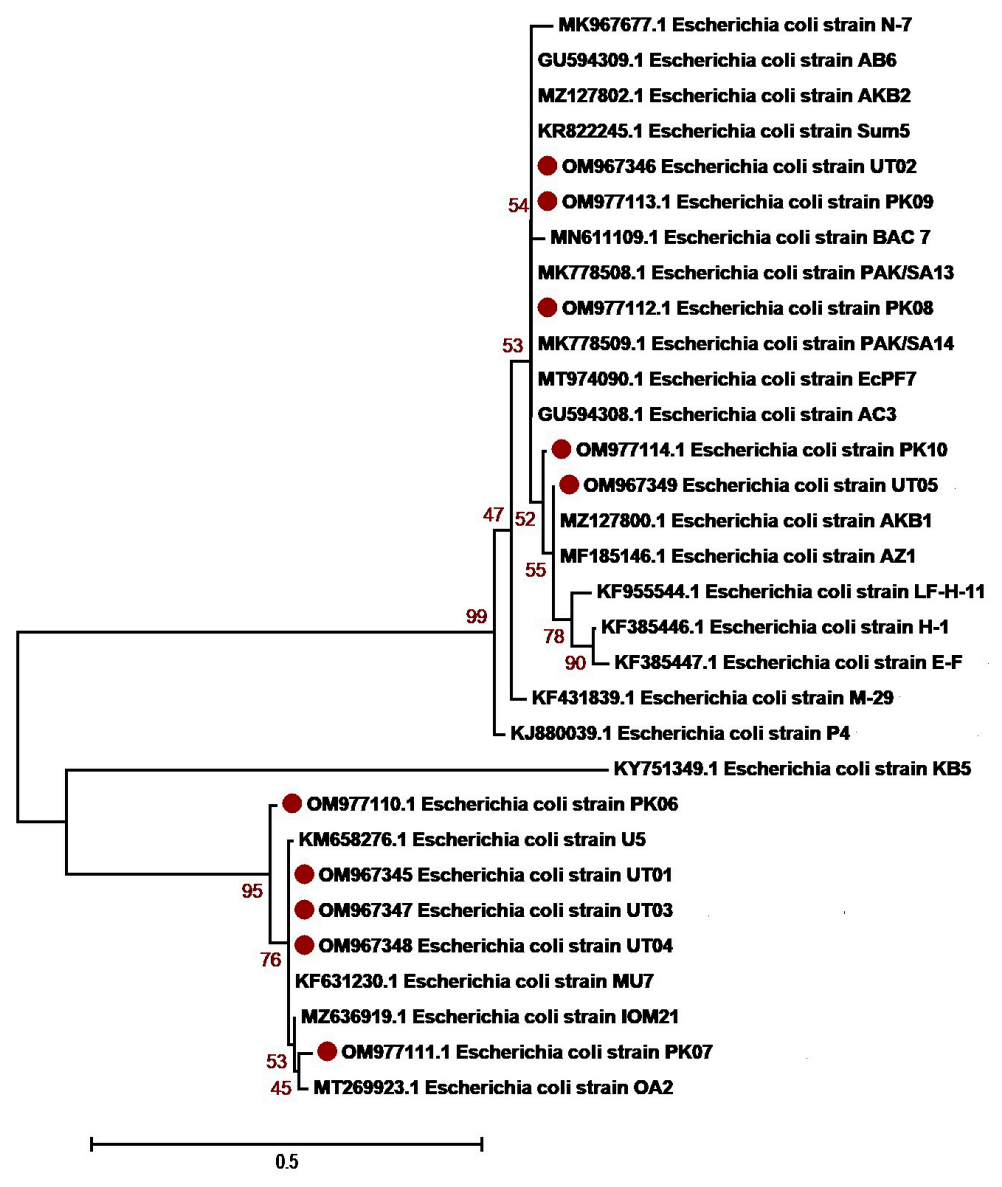
***Escherichia coli* genetic diversity based on 16 S rRNA gene sequences with reference sequences.** The analysis includes 30 nucleotide sequences from GenBank, with the accession numbers shown in parentheses. The study sequences were identified with maroon dots in the figure. The percentage of replicate trees where the related taxa clustered together in the bootstrap test (1000 repetitions). MEGA7 software was used for sequence alignment and evolutionary analyses.

**Figure 4:**
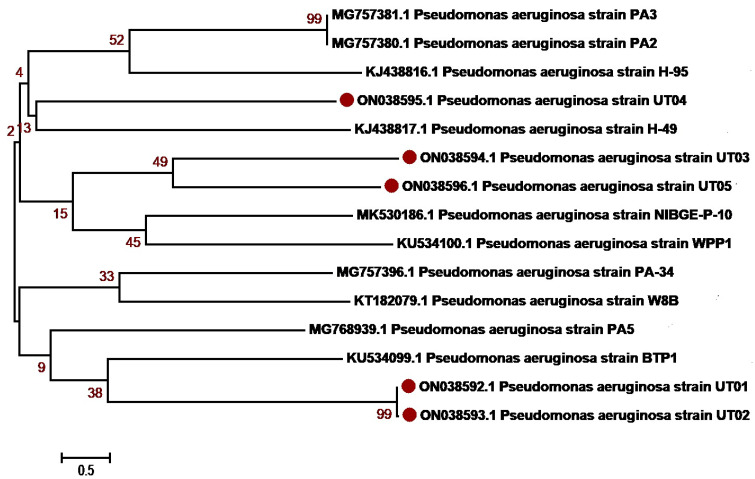
***Pseudomonas aeruginosa* genetic diversity based on 16 S rRNA gene sequences with reference sequences.** The analysis includes 15 nucleotide sequences from GenBank, with the accession numbers shown in parentheses. The study sequences were identified with maroon dots in the figure. The percentage of replicate trees where the related taxa clustered together in the bootstrap test (1000 repetitions). MEGA7 software was used for sequence alignment and evolutionary analyses.

**Figure 5:**
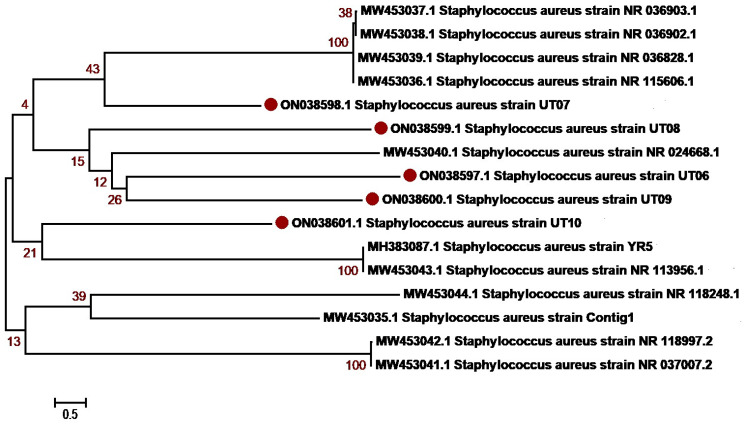
***Staphylococcus aureus* genetic diversity based on 16 S rRNA gene sequences with reference sequences.** The analysis includes 16 nucleotide sequences from GenBank, with the accession numbers shown in parentheses. The study sequences were identified with maroon dots in the figure. The percentage of replicate trees where the related taxa clustered together in the bootstrap test (1000 repetitions). MEGA7 software was used for sequence alignment and evolutionary analyses.

**Figure 6:**
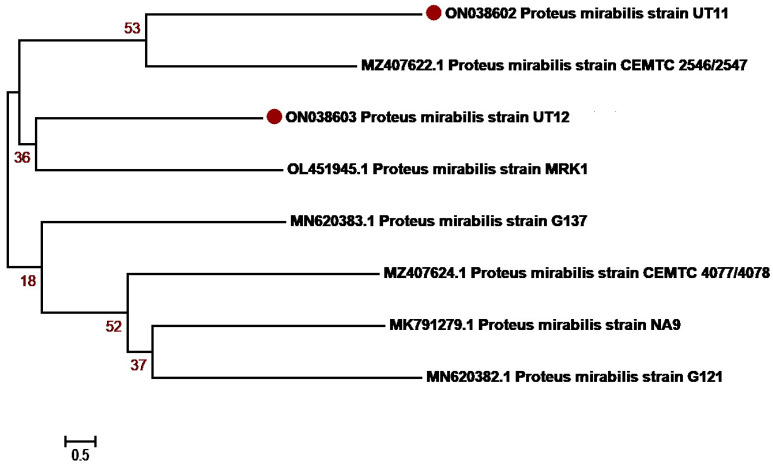
***Proteus mirabilis* genetic diversity based on 16 S rRNA gene sequences with reference sequences.** The analysis includes eight nucleotide sequences from GenBank, with the accession numbers shown in parentheses. The study sequences were identified with maroon dots in the figure. The percentage of replicate trees where the related taxa clustered together in the bootstrap test (1000 repetitions). MEGA7 software was used for sequence alignment and evolutionary analyses.

The molecular identification of the isolates was determined based on 16S rRNA sequencing. The isolates were first identified using BLAST NCBI and submitted to GenBank. Accession numbers were assigned as summarized in Table 3 and Supplementary Table S2. Sequences with a maximum percentage similarity > 99% were further subjected to phylogenetic analysis against reference sequences. Phylogenetic analysis was performed with MEGA7 using the neighbor-joining algorithm, the Kimura2 model and gamma distribution (K2 + G) and bootstrap values of 1000 repetitions (examined precision). The majority of *K. pneumoniae* isolates, OM978267, OM978269, OM978270, OM978271, OM978272, OM978273 and OM978274, from our study clustered with each other in the similar clade, indicating that most of the sequences have similarities. The remaining three sequences, OM978266, OM978268 and OM978275, clustered with other reported sequences GU594297, MN860021, MT277112 (Punjab, Pakistan) and MT271690 (Nigeria) (Figure 2). Another uropathogen, *E. coli*, showed distribution diversity. The *E. coli* isolates OM977110, OM977111, OM967345, OM967347 and OM967348 clustered in one clade with other reported sequences from Punjab Pakistan (KM658276, KF631230, MZ636919, MT269923). Other isolates, OM977112, OM977113, OM977114, OM967346 and OM967349, clustered in another clade with sequences from Pakistan (Figure 3). *P. aeruginosa*, another uropathogen detected in our study cohort, showed diversity by clustering in different clades. The isolates ON038592 and ON038593 showed similarity with KU534099 from Faisalabad, Pakistan. The isolates ON038594 and ON038596 showed sequence similarity with MK530186 and KU534100 sequences from Faisalabad, Pakistan, while another isolate, ON038595, clustered with a sequence (KJ438817) from Islamabad, Pakistan (Figure 4). *S. aureus* was detected in five patients, and their phylogenetic analysis showed diversity. The *S. aureus* isolates ON038598 clustered with other reported sequences (MW453036, MW453037, MW453038 and MW453039) from Peshawar, Pakistan. The three *S. aureus* isolates ON038597, ON038599 and ON038600 clustered in a single clade and showed similarity with MW453040 from Peshawar, Pakistan. The isolate ON038601 showed sequence homology with a reported sequence (accession number MH383087) from Islamabad, Pakistan (Figure 5). *P. mirabilis* was identified in two patients. It had high sequence similarity and clustered with sequences, as shown in Figure 6. The phylogenetic tree analysis of *K. pneumoniae*, *E. coli*, *P. aeruginosa*, *S. aureus* and *P. mirabilis* in our study clustered with each other and with the reference sequences, indicating close similarity according to the 16S rRNA method.

### 3.4. Bacterial Isolates from UTI Patients

A significant number of patient samples (*p* > 0.05) had bacterial growth (83/145; on individual plates). Gram-negative bacteria were present in 85.5% (*n* = 71/83) of the positive samples, while 14.5% (*n* = 12/83) had Gram-positive bacteria. Of the 83 confirmed bacteriuria patients, 56 (67.47%) were women and 27 (32.53%) were men. The most prevalent isolated uropathogenic bacteria was *Klebsiella pneumoniae* (33 samples, 39.7%), followed by *Escherichia coli* (23 samples, 27.7%), *Pseudomonas aeruginosa* (9 samples, 10.8%), *S. aureus* (8 samples, 9.6%), *Proteus mirabilis* (6 samples, 7.2%) and *S. saprophyticus* (4 samples, 4.8%) (Table 4, Supplementary Table S1).

### 3.5. Antibiotic Susceptibility of Gram-Negative and Gram-Positive Uropathogenic Bacteria

The sensitivities and resistance of various uropathogenic bacteria to antibiotics were examined according to the Clinical and Laboratory Standards Institute (CLSI) guidelines (Table 5). *K. pneumonia* was the most prevalent uropathogenic. It had high susceptibility to imipenem (100%) and fosfomycin (78.7%), while being resistant to cefuroxime (100%) and ciprofloxacin (94%) (Table 5). Similarly, *E. coli* showed high susceptibility to imipenem (100%), fosfomycin (78.2%) and nitrofurantoin (78.2%), while being resistant to ciprofloxacin (100%) and cefuroxime (100%). *Pseudomonas* species were sensitive to amikacin (88.8%) and imipenem (77.7%), while being resistant to cefotaxime (100%) and nitrofurantoin (100%). *Pseudomonas* had the most antibiotic resistance (Table 5). *Proteus mirabilis* was sensitive to imipenem (100%) and fosfomycin (100%), while being resistant to gentamycin (100%), cefotaxime (100%), ciprofloxacin (77.8%) and nitrofurantoin (66.7%). Gram-positive uropathogenic bacteria, *S. aureus*, showed high susceptibility to imipenem (100%) and meropenem (87.5%) and resistance to lincomycin (100%) and clindamycin (100%). *S. saprophyticus* was highly sensitive to imipenem (100%) and meropenem (75%) while being resistant to lincomycin (100%), clindamycin (100%) and ciprofloxacin (75%).

## 4. Discussion

Cases of multidrug-resistant clinical isolates are on the rise. It takes less time for isolates to become resistant to new antibiotics than to produce new antimicrobial compounds. The misuse of antibiotics in medicine, agriculture and animal husbandry has resulted in widespread antibiotic resistance. This study investigated the epidemiology and antibiotic susceptibility pattern of bacteria (Gram-negative and -positive) found in UTI patients from Lower Dir, Khyber Pakhtunkhwa (KPK), Pakistan. The study revealed that the presence of different Gram-positive and Gram-negative uropathogens, such as *S. aureus*, *S. saprophyticus*, *E. coli*, *P. aeruginosa*, *K. pneumoniae* and *Proteus mirabilis*, can cause urinary tract infections (UTIs). Previous studies have also reported that these species of bacteria can cause UTIs [42,43,44]. The results of our study concluded that female patients had higher incidences of UTIs (67.47%, 56/83) compared to male (32.53%, *n* = 12/83) patients living in the Lower Dir (Table 1). Previous studies also reported that females have higher incidences of UTIs than males within a given study population (frequency rate: 73.57% and 36.14%, respectively) [43,44,45,46]. This high incidence of UTIs in females is due to physiological changes during pregnancy, parity (number of pregnancies a person has had), hormonal changes and immune responses [47,48,49]. Our study’s results align with findings from previous studies carried out in rural and urban areas in Pakistan [50,51]. Furthermore, annual flooding events in Pakistan have left female residents in affected areas at a higher risk of contracting UTIs due to the scarcity of clean water, sanitation and worsening hygienic conditions [52,53]. The participants of our study are also residents of flood-affected areas.

The UTI rate in our study was 57.24% (*n* = 83/145), comparatively higher than that reported in a previous study (25.5%) [44]. An earlier study identified *E. coli* as the most prevalent uropathogenic (47.3%), followed by *Enterococcus faecalis* (13.6%) and *K. pneumoniae* (11.9%) [47]. However, in our study, *K. pneumoniae* was the most prevalent. Many reasons may account for the high prevalence of *K. pneumoniae* in our study cohort, such as geographic variations, antibiotic resistance and unique risk factors (compromised immune system; structural abnormalities in the urinary tract). The high prevalence of *K. pneumoniae* in patients with UTIs can be classified as healthcare-associated infections, possibly caused by prolonged hospitalization, prolonged catheterization and medical procedures. Gram-negative uropathogen frequency is 78.8%, which is significantly more than Gram-positive uropathogens [47]. Our data indicate that 85.5% of uropathogens were Gram-negative (*E. coli*, *P. aeruginosa*, *K. pneumoniae*, *Proteus mirabilis*) and that *K. pneumoniae* (39.76%) is the most prevalent, followed by *E. coli* (27.71%) (Table 4). Gram-positive uropathogens, *S. aureus* and *S. saprophyticus*, were also reported at a low frequency in our study, similar to the findings reported in previous studies [47,54,55]. The rare appearance of *S. aureus* in UTIs involves an increase in urease [55]. *S. saprophyticus* is associated with common UTIs and has a varying frequency in different communities [56,57,58]. Numerous studies have reported that multidrug-resistant *Acinetobacter baumannii* has developed into a prevalent and challenging-to-treat community- and hospital-acquired UTI [59,60,61]. However, in our study, *Acinetobacter baumannii* was absent in all patients.

This report identified bacterial isolates using various biochemical and molecular techniques. Gram staining, catalase, coagulase, oxidase, sulfide, indole and motility assessments were carried out in our study. Uropathogens were identified using Bergeys’ Manual of Systematic Bacteriology (Table 2). Several earlier studies have also used similar approaches [62,63,64]. Furthermore, the six isolates in this study were also examined via S rRNA gene sequencing. Phylogenic association was also examined to ensure that the isolates were adequately characterized. The results obtained from the BLAST analysis of target sequences showed that the input sequences had 99% similarity to the reference bacteria isolate sequences. Furthermore, the phylogenetic network analysis using the neighbor-joining method confirmed the clustering of respective bacterial isolates with their reference sequence. The results of our study are consistent with many studies showing the linkage of related bacterial isolates in the same clade [65,66].

Antibiotic resistance is a serious challenge in the treatment of UTIs. In this study, we investigated the antibiotic susceptibility pattern of bacterial isolates. Gram-negative uropathogenic bacteria were previously sensitive to imipenem (99.0%) [44]. The results of our investigation concur with this claim. However, Gram-negative bacteria in the previous study had a 98.1% susceptibility to amikacin [44], which is significantly higher than that observed in our study (52%). In another study, Gram-positive uropathogenic bacteria, *Staphylococcus aureus*, was found to be prevalent (92.3%) in Ethiopia [45], which is in contrast to the findings of our study (9.6%). In their study, *E. coli* showed high susceptibility to amikacin (82.2%), ciprofloxacin (78.2%), gentamicin (80.4%), ampicillin (59%), and nitrofurantoin (57%), while resistance to ciprofloxacin and cefuroxime was also observed [45]. In our study, *E. coli* showed high susceptibility to imipenem (100%), fosfomycin (78.2%) and nitrofurantoin (78.2%), while comparatively higher resistance to ciprofloxacin and cefuroxime was also observed. Among Gram-positive bacteria, *Staphylococcus aureus* was the most prevalent isolate, showing susceptibility to amikacin (69%) [45], which is higher than that observed in the current study (50%). Similarly, gentamicin (58.1%) and ciprofloxacin (58.4%) showed higher susceptibility compared to the observations of this study, where the susceptibility was 50% and 25%, respectively [48]. Interestingly, another similar study in Karachi, Pakistan, also revealed that Gram-negative uropathogens had the highest prevalence rate [66]. However, in that study, *E. coli* (42.9%) was found to have the highest prevalence [67], while in our study, *K. pneumoniae* (39.76%) was found to have the highest prevalence [67].

Furthermore, *P. aeruginosa* was highly susceptible to antibiotics imipenem and amikacin [49], consistent with our study’s findings. However, the prevalence of *P. aeruginosa* in Karachi was reported to be 5.4%, which is less than that reported in our study (Lower Dir, 10.8%) [68]. Therefore, the findings of this study highlight the importance of monitoring local uropathogens and susceptibility patterns. Healthcare providers should take stock of antibiotics based on local requirements. Furthermore, the results of our study showed the importance of antibiotic management and the necessity for creating new, potent antibiotics that can safely be used to treat UTIs.

## 5. Conclusions

Earlier studies have reported a plethora of findings and observations that are specific to their population of interest. Such findings have profound regional and global impacts. Our study also has similar ramifications. Specifically, the prevalence of uropathogenic bacteria was higher amongst the female population of KP, Pakistan. The incidence of UTIs was more prevalent in middle-aged individuals. *K. pneumoniae*, *E. coli*, *P. aeruginosa*, *P. mirabilis*, *S. aureus* and *S. saprophyticus* were commonly found in patient urine samples. Our phylogenetic sequence analysis further verified the identity of these bacterial isolates. It is evident that genetically varied uropathogenic organisms are widely present in UTI patients of KP. Imipenem was identified as the most effective antibiotic, followed by meropenem and fosfomycin. Interestingly, antibiotic resistance to cefuroxime was significantly high. New effective treatment regimes involving the use of the stated antibiotics may now be developed, and the spread of antibiotic resistance may be avoided. This information can contribute to our understanding of the genetic diversity and evolution of uropathogenic bacteria in Khyber Pakhtunkhwa and Pakistan. Regionally, the genetically similar populations of Afghanistan and India may also benefit from our findings. The prescription and use of antibiotics should be strictly regulated to prevent or reduce antibiotic resistance. Furthermore, whole genome sequencing and virulence gene investigations involving these uropathogens will help elaborate the role of these factors in disease severity, diagnosis and treatment.

## Figures and Tables

**Table 1:** Prevalence of UTIs in current study cohort with regard to age and gender.

Age Group	Male	Patient %	Female	Patient %	Total	Total %	*p =* Value
**5–15**	8	15.09	12	13.04	20	13.79	0.114
**16–25**	9	16.98	20	21.74	29	20	
**26–35**	5	9.43	22	23.91	27	18.62	
**36–45**	11	20.75	9	9.78	20	13.79	
**46–55**	4	7.55	11	11.96	15	10.34	
**56–65**	12	22.64	11	11.96	23	15.86	
**66–75**	4	7.55	7	7.61	11	7.59	
**Total**	53	36.55	92	63.45	145	100	

**Table 2:** Morphological and biochemical identification of uropathogens isolated from patients with urinary tract infections.

No. of Isolates	Bacteria	Colony	Gram Staining	Catalase	Coagulase	Oxidase	Sulfide	Indole	Motility
33	*K. pneumoniae*	Yellow to whitish	−ve	+ve	−ve	−ve	−ve	−ve	−ve
23	*E. coli*	Yellow opacity	−ve	+ve	−ve	−ve	−ve	+ve	+ve
09	*P. aeruginosa*	yellow, green to blue	−ve	+ve	−ve	+ve	−ve	−ve	+ve
08	*S. aureus*	Pale Yellow	+ve	+ve	+ve	−ve	−ve	−ve	−ve
06	*P. mirabilis*	Pale or colorless	−ve	+ve	−ve	−ve	+ve	−ve	+ve
04	*S. saprophyticus*	Cream to white colonies	+ve	+ve	−ve	−ve	+ve	−ve	−ve

(+ve) Positive result. (−ve) Negative result.

**Table 3:** Description of bacterial sequences submitted to NCBI GenBank with an accession number.

S. No.	Bacteria	No. of Isolates	NCBI Accession No.
1	*K. pneumoniae*	10	OM978266-OM978275
2	*E. coli*	10	OM967345-OM967349, OM977110-OM977114
3	*P. aeruginosa*	05	ON038592-ON038596
4	*S. aureus*	05	ON038597-ON038601
5	*P. mirabilis*	02	ON038602-ON038603

**Table 4:** Distribution of uropathogenic bacteria isolated from UTI patients.

S No.	Name of Pathogen	No. of Isolates	Percentage	Male		Female	
				Positive	Percentage	Positive	Percentage
**1**	*K. pneumonia*	33	39.76	11	40.74	22	39.28
**2**	*E. coli*	23	27.71	6	22.22	17	30.35
**3**	*P. aeruginosa*	9	10.84	4	14.80	5	8.92
**4**	*Proetus mirabilis*	6	7.23	2	7.40	4	7.14
**5**	*S. aureus*	8	9.64	3	11.11	5	8.92
**6**	*S. saprophyticus*	4	4.82	1	3.70	3	5.35

**Table 5:** Antibiotic susceptibility of Gram-negative and Gram-positive uropathogenic bacteria.

Antibiotics	Character	Gram-Negative Bacteria				Gram-Positive Bacteria	
		*K. pneumoniae*	*E. coli*	*P. aeruginosa*	*P. mirabilis*	*S. aureus*	*S. saprophyticus*
**Ciprofloxacin**	Susceptibility	6%	0%	22.20%	33.30%	50%	25%
	Resistance	94%	100%	77.80%	77.80%	50%	75%
**Cefuroxime**	Susceptibility	0%	0%	0%	0%	-	-
	Resistance	100%	100%	100%	100%	-	-
**Gentamicin**	Susceptibility	9%	26%	11.10%	0%	50%	50%
	Resistance	91%	74%	91.90%	100%	50%	50%
**Clindamycin**	Susceptibility	-	-	-	-	0%	0%
	Resistance	-	-	-	-	100%	100%
**Cefotaxime**	Susceptibility	33.30%	43.40%	0%	0%	-	-
	Resistance	66.70%	56.60%	100%	100%	-	-
**Fosfomycin**	Susceptibility	78.70%	78.20%	22.20%	100%	75%	50%
	Resistance	21.30%	22.80%	77.80%	0%	25%	50%
**Tazobactam**	Susceptibility	57.50%	56.50%	66.60%	50%	-	-
	Resistance	42.50%	43.50%	33.40%	50%	-	-
**Penicillin G**	Susceptibility	-	-	-	-	37.50%	50%
	Resistance	-	-	-	-	37.50%	50%
**Imipenem**	Susceptibility	100%	100%	77.70%	100%	100%	100%
	Resistance	0%	0%	22.30%	0%	0%	0%
**Cephradine**	Susceptibility	-	-	-	-	12.50%	50%
	Resistance	-	-	-	-	87.50%	50%
**Meropenem**	Susceptibility	69.60%	74%	44.40%	83.40%	87.50%	75%
	Resistance	30.40%	26%	55.60%	16.60%	12.50%	25%
**Nitrofurantoin**	Susceptibility	42.40%	78.20%	0%	33.30%	-	-
	Resistance	57.60%	21.80%	100%	66.70%	-	-
**Lincomycin**	Susceptibility	-	-	-	-	0%	0%
	Resistance	-	-	-	-	100%	100%
**Amikacin**	Susceptibility	48.40%	43.40%	88.80%	50%	75%	50%
	Resistance	51.60%	56.60%	11.20%	50%	25%	50%

## Data Availability

All data are fully available and can be found in the manuscript or in the supporting information file. Furthermore, the datasets generated or analyzed during the current study are available in the NCBI repository (https://www.ncbi.nlm.nih.gov/genbank/ (accessed on 15 January 2023)), and accession numbers (OM978266-OM978275, OM967345-OM967349, OM977110-OM977114, ON038592-ON038596, ON038597-ON038601 and ON038602-ON038603) have been provided in the Supplementary File (Table S2). Any additional information requested can be communicated with the corresponding author directly.

## Supplementary Materials

The following supporting information can be downloaded at: https://www.mdpi.com/article/10.3390/antibiotics12101508/s1, Table S1: Complete demographic characteristics of UTI patients in the current study cohort (*n* = 145); Table S2. Generated sequences from the current study submitted to NCBI GenBank, displayed with accession numbers and URLs.
