# Supplementary material for: Antibiotic Resistance Profiling and Phylogenicity of Uropathogenic Bacteria Isolated from Patients with Urinary Tract Infections
Source: Antibiotics (Basel). 2023 Oct 3;12(10):1508. doi: 10.3390/antibiotics12101508 (PMC10603882; doi:10.3390/antibiotics12101508)
Supplement: Supplementary file 1 [file antibiotics-12-01508-s001.zip › antibiotics-2572266-supplementary.pdf]

Supplementary Table S1: Complete demographic characteristics of UTI patients in the current study cohort (n=145).

S. No.	Sample ID	Age	Sex	Pathogen
1.	25691	24	F	*K. pneumoniae*
2.	29748	30	F	No growth
3.	30015	21	M	*S. aureus*
4.	31273	07	F	*E. coli*
5.	37376	41	M	*K. pneumoniae*
6.	40144	27	F	No growth
7.	41189	20	F	No growth
8.	12722	43	M	*P. aeruginosa*
9.	67387	68	F	*K. pneumoniae*
10.	16125	36	F	No growth
11.	87861	23	F	*E. coli*
12.	98154	33	F	No growth
13.	71166	14	M	No growth
14.	73572	52	F	*K. pneumoniae*
15.	76094	66	F	*P. aeruginosa*
16.	57177	26	F	No growth
17.	48349	63	M	*K. pneumoniae*
18.	30139	50	F	No growth
19.	05798	34	M	*K. pneumoniae*
20.	23075	24	F	No growth
21.	58864	39	F	*E. coli*
22.	74008	35	F	No growth
23.	55687	19	F	No growth
24.	26147	53	M	*E. coli*
25.	36679	34	F	No growth
26.	43166	31	F	*S. aureus*
27.	35605	40	M	No growth
28.	88347	72	F	*K. pneumoniae*
29.	84164	65	F	*E. coli*
30.	41865	05	F	*E. coli*
31.	67211	38	M	*P. mirabilis*
32.	89627	21	F	No growth
33.	11427	25	F	No growth
34.	19496	57	M	*K. pneumoniae*
35.	65941	61	F	*P. mirabilis*
36.	69162	19	F	No growth
37.	79081	12	M	No growth
38.	84268	27	M	*P. aeruginosa*

39.	80322	16	F	*K. pneumoniae*
40.	21117	56	F	No growth
41.	14869	25	F	No growth
42.	21171	43	M	*K. pneumoniae*
43.	89999	24	M	*E. coli*
44.	47159	59	F	No growth
45.	41317	35	M	No growth
46.	99039	09	F	*K. pneumoniae*
47.	93089	23	M	No growth
48.	99251	65	M	*S. saprophyticus*
49.	39364	36	F	*E. coli*
50.	84256	27	F	No growth
51.	92384	33	F	*K. pneumoniae*
52.	44689	75	M	*S. aureus*
53.	30272	14	M	No growth
54.	88012	45	F	No growth
55.	47359	47	M	*S. saprophyticus*
56.	47939	20	F	No growth
57.	82332	67	F	*K. pneumoniae*
58.	58474	14	F	*K. pneumoniae*
59.	80821	46	F	No growth
60.	13636	17	F	*E. coli*
61.	64309	20	M	No growth
62.	90441	55	F	*P. aeruginosa*
63.	80507	15	F	No growth
64.	10796	54	F	*K. pneumoniae*
65.	19104	43	F	*S. aureus*
66.	93543	36	M	No growth
67.	13411	56	M	No growth
68.	73943	23	F	No growth
69.	32929	64	F	*S. saprophyticus*
70.	77807	65	M	*K. pneumoniae*
71.	44209	15	M	*E. coli*
72.	36133	44	M	*P. aeruginosa*
73.	63898	14	F	No growth
74.	14833	72	M	*K. pneumoniae*
75.	60691	22	F	*E. coli*
76.	35118	26	F	No growth
77.	47838	23	F	*K. pneumoniae*
78.	89857	52	F	No growth
79.	47082	34	F	*S. aureus*

80.	90487	21	M	*S. aureus*
81.	48096	59	F	*E. coli*
82.	77083	35	F	*K. pneumoniae*
83.	32247	19	F	No growth
84.	35252	61	F	*K. pneumoniae*
85.	20985	34	F	*P. mirabilis*
86.	88991	31	F	*K. pneumoniae*
87.	48938	40	F	*E. coli*
88.	49238	70	F	*P. aeruginosa*
89.	76813	65	M	*P. mirabilis*
90.	56167	11	F	No growth
91.	99709	38	M	*E. coli*
92.	77456	29	F	*E. coli*
93.	56227	14	M	No growth
94.	30803	53	F	*K. pneumoniae*
95.	83688	61	M	No growth
96.	64326	25	F	No growth
97.	92778	31	F	No growth
98.	52962	56	M	*K. pneumoniae*
99.	67724	62	F	No growth
100.	17518	33	M	*E. coli*
101.	73856	68	F	*E. coli*
102.	60675	13	M	No growth
103.	64958	45	F	*S. saprophyticus*
104.	77102	47	M	*K. pneumoniae*
105.	76686	20	F	No growth
106.	65578	74	M	No growth
107.	85147	14	F	*K. pneumoniae*
108.	23535	46	F	*K. pneumoniae*
109.	93032	17	F	*P. mirabilis*
110.	74825	29	F	*E. coli*
111.	93225	55	F	*P. aeruginosa*
112.	97028	14	M	No growth
113.	90564	34	F	No growth
114.	50956	43	M	*K. pneumoniae*
115.	67802	36	M	No growth
116.	66925	56	F	*K. pneumoniae*
117.	58583	23	M	No growth
118.	55217	58	F	*E. coli*
119.	93767	65	M	No growth
120.	60596	15	F	*S. aureus*

121.	38556	44	M	No growth
122.	97519	14	F	*K. pneumoniae*
123.	40241	71	M	No growth
124.	38011	48	F	*E. coli*
125.	20191	24	F	No growth
126.	13702	53	M	No growth
127.	11733	34	F	*K. pneumoniae*
128.	16512	31	F	*E. coli*
129.	97458	40	F	*K. pneumoniae*
130.	62118	69	F	*P. aeruginosa*
131.	46499	65	M	No growth
132.	93254	09	M	*E. coli*
133.	82746	58	M	No growth
134.	78028	29	F	No growth
135.	42221	15	F	No growth
136.	12131	53	M	*P. aeruginosa*
137.	42438	61	M	*K. pneumoniae*
138.	72671	25	M	No growth
139.	39514	10	F	No growth
140.	43371	31	F	*E. coli*
141.	49716	27	M	No growth
142.	47675	20	M	No growth
143.	29694	43	F	*P. mirabilis*
144.	68225	65	F	*K. pneumoniae*
145.	78954	23	F	*S. aureus*

Table S2. Generated sequences from the current study submitted to NCBI GenBank, displayed with accession numbers and URLs.

S. No.	Bacteria	Accession Number	URLs
1	*K. pneumoniae*	OM978266	https://www.ncbi.nlm.nih.gov/nuccore/OM978266
2	*K. pneumoniae*	OM978267	https://www.ncbi.nlm.nih.gov/nuccore/OM978267
3	*K. pneumoniae*	OM978268	https://www.ncbi.nlm.nih.gov/nuccore/OM978268
4	*K. pneumoniae*	OM978269	https://www.ncbi.nlm.nih.gov/nuccore/OM978269
5	*K. pneumoniae*	OM978270	https://www.ncbi.nlm.nih.gov/nuccore/OM978270
6	*K. pneumoniae*	OM978271	https://www.ncbi.nlm.nih.gov/nuccore/OM978271
7	*K. pneumoniae*	OM978272	https://www.ncbi.nlm.nih.gov/nuccore/OM978272
8	*K. pneumoniae*	OM978273	https://www.ncbi.nlm.nih.gov/nuccore/OM978273
9	*K. pneumoniae*	OM978274	https://www.ncbi.nlm.nih.gov/nuccore/OM978274
10	*K. pneumoniae*	OM978275	https://www.ncbi.nlm.nih.gov/nuccore/OM978275
11	*E. coli*	OM967345	https://www.ncbi.nlm.nih.gov/nuccore/OM967345
12	*E. coli*	OM967346	https://www.ncbi.nlm.nih.gov/nuccore/OM967346
13	*E. coli*	OM967347	https://www.ncbi.nlm.nih.gov/nuccore/OM967347
14	*E. coli*	OM967348	https://www.ncbi.nlm.nih.gov/nuccore/OM967348
15	*E. coli*	OM967349	https://www.ncbi.nlm.nih.gov/nuccore/OM967349
16	*E. coli*	OM977110	https://www.ncbi.nlm.nih.gov/nuccore/OM977110
17	*E. coli*	OM977111	https://www.ncbi.nlm.nih.gov/nuccore/OM977111
18	*E. coli*	OM977112	https://www.ncbi.nlm.nih.gov/nuccore/OM977112
19	*E. coli*	OM977113	https://www.ncbi.nlm.nih.gov/nuccore/OM977113
20	*E. coli*	OM977114	https://www.ncbi.nlm.nih.gov/nuccore/OM977114
21	*P. aeruginosa*	ON038592	https://www.ncbi.nlm.nih.gov/nuccore/ON038592
22	*P. aeruginosa*	ON038593	https://www.ncbi.nlm.nih.gov/nuccore/ON038593
23	*P. aeruginosa*	ON038594	https://www.ncbi.nlm.nih.gov/nuccore/ON038594
24	*P. aeruginosa*	ON038595	https://www.ncbi.nlm.nih.gov/nuccore/ON038595
25	*P. aeruginosa*	ON038596	https://www.ncbi.nlm.nih.gov/nuccore/ON038596
26	*S. aureus*	ON038597	https://www.ncbi.nlm.nih.gov/nuccore/ON038597
27	*S. aureus*	ON038598	https://www.ncbi.nlm.nih.gov/nuccore/ON038598
28	*S. aureus*	ON038599	https://www.ncbi.nlm.nih.gov/nuccore/ON038599
29	*S. aureus*	ON038600	https://www.ncbi.nlm.nih.gov/nuccore/ON038600
30	*S. aureus*	ON038601	https://www.ncbi.nlm.nih.gov/nuccore/ON038601
31	*P. mirabilis*	ON038602	https://www.ncbi.nlm.nih.gov/nuccore/ON038602
32	*P. mirabilis*	ON038603	https://www.ncbi.nlm.nih.gov/nuccore/ON038603
